# Rethinking Data Sharing at the Dawn of a Health Data Economy: A Viewpoint

**DOI:** 10.2196/11519

**Published:** 2018-11-22

**Authors:** Chunlei Tang, Joseph M Plasek, David W Bates

**Affiliations:** 1 Division of General Internal Medicine and Primary Care Brigham and Women’s Hospital Boston, MA United States; 2 Harvard Medical School Harvard University Boston, MA United States; 3 Clinical and Quality Analysis Partners HealthCare System Boston, MA United States; 4 Department of Biomedical Informatics School of Medicine University of Utah Salt Lake City, UT United States

**Keywords:** economics, hospital, machine learning, models, economic, precision medicine

## Abstract

A health data economy has begun to form, but its rise has been tempered by the profound lack of sharing of both data and data products such as models, intermediate results, and annotated training corpora, and this severely limits the potential for triggering economic cluster effects. Economic cluster effects represent a means to elicit benefit from economies of scale from internal data innovations and are beneficial because they may mitigate challenges from external sources. Within institutions, data product sharing is needed to spark data entrepreneurship and data innovation, and cross-institutional sharing is also critical, especially for rare conditions.

Data innovation and data entrepreneurship have the potential to dramatically alter the current health care landscape as health data economy is beginning to revolutionize the field [1-3]. The European Commission estimated that the value of the European Union data economy would increase to US $860 (€739) billion by 2020, up from US $331 (€285) billion in 2015 [4,5]. Data economy, wherein health care will increasingly participate, has formed, and it is lucrative and quickly growing. Sharing data is necessary to enable thriving health data economy and produce clinical advances that are not possible in the current health care environment because of siloed data resources. These data resources span from the bench to the bedside and beyond, including genetic, genomic, proteomic, clinical, imaging, patient-centered, public health, and other relevant data. Electronic health record systems enable health care organizations to share clinical data across their organization, with patients themselves through patient portals, and to a limited extent owing to a lack of interoperability, with other organizations or systems. Rethinking how we share data and data products is essential for health data economy to thrive.

Data products, such as models, intermediate results, and annotated training corpora, are the outcomes from data preparation, processing, and analysis (eg, statistical analysis, data mining, and machine or deep learning). Data products also include visualizations and dashboards created by the artistic manual work of data scientists to assist in the interpretation of the analysis in an actionable way. Data products, like data itself, are “nonrivalrous,” meaning that they can be utilized by >1 data scientist at a time to create additional data products or services. For example, critical to the development of deep neural networks for image recognition tasks is the training set of >10,000 labeled images on ImageNet [6] created by manual annotation efforts that were made publicly available. Similarly, raw journal article titles can be easily searched through PubMed or MEDLINE, yet a data product from this resource that is created after standard text processing techniques (eg, tokenization and stop-word removal) have been applied is usable for many subsequent analyses. However, similar data products at scale tend to not be available in health care, resulting in a lack of generalizability for models and concerns regarding the reproducibility of results.

Sharing data products across health care provider networks can reveal different insights into different clinical departments and may also indirectly promote the core business of health care through better revenue and profitability margins, as data products can easily be used for secondary purposes. The second benefit of data sharing is to allow data to spread beyond the current data silos, which would facilitate data entrepreneurship, data innovation, data processing, and secondary data mining.

Data products need not contain identifiable patient data that would be useful for general research purposes. Deidentified data products from clinical care must be treated with appropriate care and respect. If one had a covariance matrix and corresponding mean vector for variables, one could run regression or advanced analyses using structural equation modeling to explore latent variables that were not even postulated in the original research. The National NLP Clinical Challenges [7] provides annotated, fully deidentified corpora of clinical notes centering around particular clinical tasks, allowing researchers to start with a verified gold standard and benchmark their systems against others. As the Medical Information Mart for Intensive Care III [8] contains both structured and unstructured data and is accessible to researchers, any data products (eg, annotated clinical notes and models) built on top of this or similar resources, should they be made available, could be openly critiqued and improved upon by the community.

Learning health care systems and precision medicine are two data-driven innovations at different scales in the health care data environment, where sharing data and data products are most applicable. Learning health systems are centered on the organization where new knowledge is captured as an integral byproduct of the delivery experience [9]. For example, electronic health record data that contain rich clinical information (eg, patients’ medical history, family history, surgical resection approach, and postoperative supervision) offer an opportunity to design algorithms for acute interventions, such as predicting 30-day hospital readmission or whether a patient is at risk for cardiac decompensation. Similarly, exploring care process protocols, including a combination of medications, for a specific disease could inform drug inventory management. Precision medicine represents a leading driver of the health data economy in which health care recommendations can be individually tailored on the basis of a person’s genes, lifestyle, and environment [10]. Similarity-based classifiers aimed at automatically grouping patients with similar characteristics together enable improvements in assessment, diagnosis, the selection of therapeutic choice, and the prediction of prognosis. For example, abnormalities in a clinical pathway could be highlighted using trend recognition algorithms to identify a similarity cohort to allow the assessment of the complexity associated with a disease cluster. Furthermore, sharing data is critical for rare diseases, both from a learning health care system perspective to optimize the delivery of care and a precision medicine perspective to be able to effectively personalize the care plan.

We envision that economic cluster effects (ie, a geographic concentration of interconnected stakeholders and their associated institutions in a field through a nested interorganizational network of relationships) within the health data economy will emerge soon, but that the sharing of data products will be necessary to maximize their potential. Multistakeholder health data governance would be beneficial, as it would allow balancing of value for all actors (eg, clinicians, patients, and other data generators; data scientists, researchers, and other data product enhancers), which is useful in determining not only how data products should be owned but also what types of data should be shared to maximize data resource utilization toward the problems of interest to the community. The status quo is far from optimal from an economic perspective, and we collectively have poorer health [11] because of this lack of sharing and void in meaningful governance. From a technical perspective, blockchain or similar technologies can be utilized to insure the integrity of shared data and data products. Only with the wide availability and use of diverse data products will the future of learning health systems and precision medicine be truly accessible in the emerging health data economy.
